# Obesity and Its Relationship with Hypertension among Adults 50 Years and Older in Jinan, China

**DOI:** 10.1371/journal.pone.0114424

**Published:** 2014-12-17

**Authors:** Shu-Kang Wang, Wei Ma, Shumei Wang, Xiang-Ren Yi, Hong-Ying Jia, Fuzhong Xue

**Affiliations:** 1 Department of Epidemiology and Biostatistics, School of Public Health, Shandong University, Jinan, China; 2 The College of Physical Education of Shandong University, Jinan, China; 3 The Second Hospital of Shandong University, Jinan, China; St. Michael's hospital, Canada

## Abstract

**Background:**

The relationship between obesity and hypertension varies with geographical area, race and definitions of obesity. Our study aimed to investigate the prevalence of obesity using standard Chinese criteria based on the body mass index (BMI) and the waist circumference (WC) and to examine the association between obesity and hypertension among middle-aged and elderly people in Jinan city.

**Methods:**

This cross-sectional study examined 1,870 subjects from the blocks randomly selected from among the 6 communities of Jinan, China in 2011–2012. The Student's t-test was used to compare numerical data, and the χ^2^ test was used to compare categorical data. Multivariate logistic regression analyses were performed to assess the effects of general and central obesity on hypertension after adjusting for age or for education level, smoking, alcohol consumption, and continuous age.

**Results:**

The prevalence of general obesity among people age 50 years and older was 21.1% (17.0% for males and 23.1% for females), and the prevalence of central obesity was 77.8% for men and 78.7% for women. For men, compared with a normal BMI, the ORs and 95% CIs for overweight and general obesity were 1.853 (1.252, 2.744) and 3.422 (1.894, 6.182), respectively, after adjusting for age, smoking, alcohol consumption and educational level. Compared with a normal WC, the ORs and 95% CIs for central obesity were 2.334 (1.573, 3.465) and 2.318 (1.544, 3.479), respectively, for men. For women, compared with a normal BMI, the ORs and 95% CIs were 1.942 (1.473, 2.599) and 4.011 (2.817, 5.712), respectively, after adjusting for age, smoking, alcohol consumption and educational level. Compared with a normal WC, the ORs and 95% CIs for central obesity were 2.488 (1.865, 3.319) and 2.379 (1.773, 3.192), respectively, for women.

**Conclusions:**

The relationship between hypertension and general obesity was stronger than the relationship between hypertension and either overweight or central obesity in both genders.

## Introduction

It is well known that obesity is one of the most important public health problems worldwide [1]. It is a major independent risk factor for chronic diseases such as cardiovascular disease (CVD) and diabetes mellitus; obesity is also associated with high morbidity and mortality [2], [3]. Over the past 20 years, the prevalence of obesity has increased greatly worldwide [4] and in China, in particular [5]. A relationship between obesity, including both general obesity and central obesity, and hypertension has been suggested in many studies [6]–[9]. Two of the most commonly used anthropological indices in clinical practice and population surveys are the body mass index (BMI) and the waist circumference (WC), indicating general obesity and central obesity, respectively.

Criteria for obesity have been written in terms of both the BMI and the WC; in other words, the relationship between obesity and hypertension depends on the particular standard of obesity. Anthropometric indices vary greatly across different ethnic groups [10]–[13]; although the standard international criteria for the measurement of body fat based on the BMI and the WC were defined by the World Health Organization (WHO) [14], these criteria are suitable for people of European descent but not of Chinese descent. Thus, an obesity task force in China generated standard criteria for overweight and obesity in China based on BMI and WC values appropriate for Chinese people [15], [16]. Due to China's vast size and large population, the prevalences of overweight and obesity [17] varies substantially within the country; in particular, the prevalence is higher in northern China than in southern China. The population in northern China is taller and heavier, and they have higher BMIs and WCs compared with the southern population [18]. Thus, the relationship between obesity and hypertension varies with age, race, geographical region and the different obesity standards that are used.

To date, few studies have examined the relationship between obesity and hypertension among middle-aged and elderly people in the northern Chinese urban communities according to the Chinese standard criteria for obesity. Thus, the aims of our study were to investigate the prevalence of obesity according to the Chinese standard criteria based on the BMI and the WC and to examine the associations between obesity and hypertension for middle-aged and elderly people in a community of Jinan city.

## Materials and Methods

### Subjects

The subjects were from the blocks randomly selected from among the 6 communities of Jinan, China in 2011–2012. The inclusion criteria for the participants included: (1) age≥50 years, (2) ability to answer the questionnaire, and (3) living in the selected communities for more than 6 months in the past year. A total of 3,277 residents aged 50 years or older were potentially eligible for this study; however, 1,407 individuals were excluded because they did not provide anthropometric data such as their height, weight, WC, systolic blood pressure (SBP), diastolic blood pressure (DBP), and information about their current use of medications. A total of 1,870 participants were included in the final data analysis.

### Investigation and measurements

Trained interviewers administered a standardized questionnaire to obtain demographic information including subjects'age, sex, educational attainment (uneducated, grade school, junior high school, senior high school, junior college or college and above), smoking status (never, current or former), and alcohol consumption (never, current or former).

All participants were given a standardized medical examination in which their systolic blood pressure (SBP) and diastolic blood pressure (DBP) were measured. Blood pressure (BP) was measured in the right arm by trained examiners using a mercury sphygmomanometer according to a standard protocol [19]. Three BP measurements were made, with an interval of from 5 to 15 minutes between measurements, and the average of the three readings was chosen as the BP value for each subject. The anthropometric measurements were taken after the participants had removed their shoes and any heavy clothing or belts. Each subject's height, weight, and WC were measured by trained nurses. The WC was measured at the level midway between the lower rib margin and the iliac crest while the participants breathed out gently. This study was approved by the Ethics Committee of the School of Public Health of Shandong University, and written informed consent of each participant was obtained.

### Definition of obesity and hypertension

For each subject, the BMI was calculated as weight (kg)/height^2^ (m^2^). The Chinese standard of general obesity, defined in terms of the BMI, was used in this study. Subjects' weights were classified according to the standard criteria for China set forth by the Chinese Obesity Working Group: BMI<18.5: underweight; 18.5≤BMI<24: normal; 24≤BMI<28: overweight; and BMI≥28: general obesity [20], [21]. Central obesity was defined as a WC≥85 cm for males and a WC≥80 cm for females [22]. The presence of hypertension was defined by an SBP≥140 mmHg, a DBP≥90 mmHg or the self-reported current use of antihypertensive medications.

### Statistical analysis

Descriptive statistics were generated for all variables, according to gender. Numerical data were expressed as the mean and standard deviation (mean ±s.d.), and categorical data were expressed as percentages. The t-test was used to examine differences in the means of variables (age, height, weight, BMI, WC, SBP and DBP) between study groups, and the χ^2^ test was used to compare the prevalences of general obesity, central obesity, hypertension, smoking and alcohol consumption between males and females. The differences in BMI and WC across age groups were described by sex, and the χ^2^ test was used to compare the prevalence of obesity between different age groups in the study population. The Bonferroni correction was used for multiple comparisons. Descriptive analyses of the characteristics of the hypertensive and normotensive groups were performed, and the characteristics of these groups were compared. Logistic regression analysis was used to determine odds ratio (OR) values with 95% confidence intervals (CIs) for hypertension in the study population. Two logistic regression models were generated. In the first model, only continuous age was adjusted for, and in the second model, education level (uneducated, grade school, junior high school, senior high school, junior college, and college and above), smoking (never, current or former), alcohol consumption (never, current or former), and continuous age were adjusted for. Categorical BMI and WC values were the independent variables in models one and two, respectively. SPSS16.0 was used to perform the t-tests, χ^2^ tests and logistic regression analyses.

## Results

A total of 1,870 people (600 men and 1,270 women) age 50 years and older participated in this study. The distributions of age, height, weight, BMI, WC, SBP and DBP by gender and comparisons between males and females are summarized in Table 1. The mean age was 64.76±9.09 years for men and 64.45±9.67 years for women; no significant difference in age was found between males and females (P = 0.494). The prevalences of hypertension, smoking, alcohol consumption, general obesity calculated in terms of the BMI, central obesity calculated in terms of the WC and each educational level are also listed in Table 1. The results show that height, weight, WC, DBP, the prevalences of general obesity, hypertension, smoking, alcohol consumption and all education levels except the junior high school level were significantly different between males and females (P<0.05).

**Table 1:** Summary statistics and a comparison of characteristics between genders (mean ± s.d.).

Characteristic	Total (n = 1870)	Male (n = 600)	Female(n = 1270)	P
Age (year)	64.55±9.49	64.76±9.09	64.45±9.67	0.494
Height (cm)	160.96±8.12	169.10±5.88	157.11±5.88	<0.001
Weight (kg)	65.97±10.92	72.35±10.23	62.96±9.89	<0.001
BMI	25.43±3.56	25.27±3.13	25.50±3.74	0.174
WC (cm)	88.72±10.13	91.34±9.30	87.48±10.27	<0.001
SBP (mmHg)	135.04±19.66	135.58±19.08	134.78±19.93	0.410
DBP (mmHg)	83.44±11.44	85.09±11.46	82.66±11.36	<0.001
General obesity, n (%)	395 (21.1)	102 (17.0)	293 (23.1)	0.003
Central obesity, n (%)	1467 (78.4)	467 (77.8)	1000 (78.7)	0.673
Hypertension, *n* (%)	1149 (61.4)	400 (66.7)	749 (59.0)	0.001
Smoking, n (%)	284 (15.2)	236 (39.3)	48 (3.8)	<0.001
Alcohol drinking (%)	216 (11.6)	190 (31.7)	26 (2.0)	<0.001
Educational attainment*				
Uneducated (%)	247 (13.3)	16 (2.7)	231 (18.3)	<0.001
Grade school (%)	348 (18.7)	83 (13.9)	265 (20.9)	<0.001
Junior high school (%)	469 (25.2)	153 (25.7)	316 (25.0)	0.749
Senior high school (%)	629 (33.8)	239 (40.1)	390 (30.8)	<0.001
Junior college and above (%)	168(9.0)	105(17.6)	63 (5.0)	<0.001

BMI, body mass index; WC, waist circumference; SBP, systemic blood pressure; DBP, diastolic blood pressure.

*There were four missing values for men and five missing values for women for educational attainment.

Table 2 presents the prevalences of general obesity, central obesity and hypertension for each age group. For males, significant differences in the prevalences of general obesity, central obesity and hypertension were not found between age groups (P>0.05). For females, a significant difference in the prevalence of general obesity was found only between the age 50–59 group and the age 70–79 group (P = 0.001); however, significant differences in the prevalence of central obesity and hypertension were found between the age 50–59 group and the other age groups (P<0.001), and a significant difference in the prevalence of hypertension was also found between the age 60–69 group and the age 70–79 group (P<0.001).

**Table 2:** Prevalences of general obesity and central obesity in the study population.

Age (y)	Male (%)				Female (%)			
	n	GO	CO	HP	n	GO	CO	HP
50–59	210	40(19.0)	167(79.5)	134(63.8)	489	93(19.0)	327(66.9)	223(45.6)
60–69	214	33(15.4)	157(73.4)	147(68.7)	367	86(23.4)	305(83.1)	216(58.9)
70–79	124	21(16.9)	100(80.6)	84(67.7)	323	95(29.4)	286(88.5)	243(75.2)
≥80	52	8(15.4)	43(82.7)	35(67.3)	91	19(20.9)	82(90.1)	67(73.6)
P	-	>0.05	>0.05	>0.05	-	<0.05	<0.05	<0.05

GO: general obesity; CO: central obesity; HP: hypertension.

The distributions of age, height, weight, BMI and WC between the hypertensive and normotensive groups are shown in Table 3. The prevalences of general obesity, central obesity, smoking and alcohol consumption are also listed in Table 3. All variables were significantly different between the hypertensive and normotensive groups except the average height and the prevalence of smoking.

**Table 3:** Summary statistics and a comparison of characteristics between the hypertensive and normotensive groups (mean ±s.d.).

Characteristics	Hypertensive (n = 1149)	Normotensive (n = 721)
Age (year)	66.01±9.48*	62.23±9.04*
Height (cm)	161.03±8.45	160.84±7.58
Weight (kg)	67.80±11.00*	63.07±10.13*
BMI	26.11±3.57*	24.34±3.27*
WC(cm)	90.91±9.71*	85.22±9.80*
General obesity, n (%)	309(26.9)*	86(11.9)*
Central obesity, n (%)	980(85.3)*	487(67.5)*
Smoking, n (%)	179(15.6)	105(14.6)
Alcohol consumption (%)	148(12.9)*	68(9.4)*

BMI, body mass index; WC, waist circumference;

*P<0.05.

Table 4 shows the association between obesity and hypertension in both genders. In males, compared with a normal BMI, the ORs and 95% CIs for overweight and general obesity were 1.843(1.258, 2.700) and 3.632(2.031, 6.492), respectively, when adjusting for age, and the ORs and 95% CIs were 1.853(1.252, 2.744) and 3.422(1.894, 6.182), respectively, when adjusting for age, smoking, alcohol consumption and educational level. Compared with a normal WC, the ORs and 95% CIs for central obesity were 2.334(1.573, 3.465) and 2.318(1.544, 3.479), respectively. In females, compared with a normal BMI, the ORs and 95% CIs for overweight and general obesity were 1.962(1.495, 2.574) and 4.061(2.870, 5.745), respectively, when adjusting for age, and the ORs and 95% CIs were 1.942(1.473, 2.599) and 4.011(2.817, 5.712), respectively, when adjusting for age, smoking, alcohol consumption and educational level. Furthermore, compared with a normal WC, the ORs and 95% CIs for central obesity were 2.488(1.865, 3.319) and 2.379(1.773, 3.192), respectively.

**Table 4:** Association of hypertension with BMI and WC by gender.

Gender	N (%)	Hypertension		
		N (%)	OR*(95%CI)	OR**(95%CI)
Males	600	400(66.7)		
BMI(kg/m^2^)				
BMI<18.5	9(1.5)	4(44.4)	0.608(0.157,2.354)	0.748(0.189, 2.959)
18.5≤BMI<24	178(29.7)	98(55.1)	1.0	1.0
24≤BMI<28	311(51.8)	215(69.1)	1.843(1.258,2.700)	1.853(1.252,2.744)
BMI≥28	102(17.0)	83(81.4)	3.632(2.031,6.492)	3.422(1.894, 6.182)
WC(cm)				
WC<85	133(22.2)	68(51.1)	1.0	1.0
WC≥85	467(77.8)	332(71.1)	2.334(1.573,3.465)	2.318(1.544,3.479)
Females	1270	749(59.0)		
BMI(kg/m^2^)				
BMI<18.5	31(2.4)	12(38.7)	0.621(0.283,1.361)	0.655(0.295,1.453)
18.5≤BMI<24	400(31.5)	178(44.5)	1.0	1.0
24≤BMI<28	546(43.0)	333(61.0)	1.962(1.495,2.574)	1.942(1.473,2.599)
BMI≥28	293(23.1)	226(77.1)	4.061(2.870,5.745)	4.011(2.817,5.712)
WC(cm)				
WC<80	270(21.3)	101(37.4)	1.0	1.0
WC≥80	1000(78.7)	648(64.8)	2.488(1.865,3.319)	2.379(1.773,3.192)

*adjusted for age,

**adjusted for age, education level, smoking and alcohol consumption.

## Discussion

The prevalence of obesity has increased worldwide and has nearly doubled between 1980 and 2008 [23]. A large number of studies have shown that the risk of obesity increases in those with hypertension, and the relationship between obesity and hypertension differs according to age, gender, geographical area and race [24], [25]. Thus, in this study, we examined the relationship between obesity and hypertension among people over 50 years of age in Jinan, China. We showed that height, weight, WC, DBP, and the prevalences of general obesity, hypertension, smoking and alcohol consumption were significantly different between males and females, suggesting that gender is a strong confounder. Then subgroup analyses were conducted in males and females in order to intuitively to show and compare the relationships between obesity and hypertension. Perhaps because the women were more willing to comply with the requirements of the survey and the physical examination than men, our final sample included more women than men.

Estimates of the prevalence of obesity were obviously related to the definition of obesity. The WHO defines the cutoff BMI values for overweight and general obesity for Caucasian populations as 25 and 30, respectively [26]. Previous studies have shown that Chinese and many other Asian populations have a lower BMI but a higher percentage of body fat than Caucasians of a similar age and sex [27], [28]. Thus, criteria for obesity in the Chinese population were established by the Chinese Obesity Working Group. In this study, using the standard criteria set forth by the Chinese Obesity Working Group, the prevalence of general obesity among people age 50 years and over was 21.1% (17.0% in males and 23.1% in females), and the prevalence of general obesity in females was significantly higher than that in males. Our results regarding the prevalence of general obesity are consistent with previous findings in other Han Chinese populations [29]. The prevalence of central obesity was 77.8% for men and 78.7% for women, both of which were significantly higher than prevalences reported in previous studies [30]; this difference may have arisen because only an urban population was included in this study. Many studies have reported that the prevalence of obesity among urban residents is higher than that among rural residents in China [31], [32], and Jinan, as a provincial capital city, has a relatively high urbanization level; thus, in our study, the prevalence of central obesity was found to be higher than that reported in previous studies.

The prevalence of obesity in this study differed between males and females according to age group. In males, no significant difference in the prevalence of obesity was found between age groups. In females, the prevalence of general obesity increased with increasing age and was highest in the age 70–79 group; a significant difference in the prevalence of general obesity was only found between the age 50–59 group and the age 70–79 group. However, significant differences in the prevalence of central obesity were found between the age 50–59 group and all the other age groups, but no significant difference was found between the other age groups. These results suggest that the sixth and seventh decades of life are critical to the development of central obesity for females; this conclusion has been confirmed in other studies, as well [33]. Thus, we should pay attention to changes in body fat distribution that lead to an increased risk of cardiovascular and metabolic diseases in menopause [34].

Hypertension is closely associated with obesity [35], [36], and in this study we confirmed the correlations between hypertension and overweight, general obesity and central obesity. The relationship between obesity and hypertension in our study can be summarized as follows. First, overweight, general obesity and central obesity were all positively correlated with hypertension in both genders. Second, the relationship between hypertension and general obesity was stronger than the relationship between hypertension and either overweight or central obesity in both genders, but the only significant difference in ORs was found between overweight and general obesity for females. The results of our study were similar to those of a previous large population survey conducted in China [37]. Using the standard criteria for China set by the Chinese Obesity Working Group, the association between hypertension and general obesity was found to be stronger than that between hypertension and central obesity.

Our investigation has several limitations. First, our study had a cross-sectional design, which can be used to explore the associations between obesity and hypertension but cannot be used to explore causation. To overcome this limitation, it would be useful to conduct a prospective cohort study in the future. Second, approximately half the sample in this study did not take part in the physical examination, and this circumstance may have created a selection bias.

In conclusion, hypertension is more strongly correlated with general obesity than with central obesity in adults 50 years of age and older in Jinan, China. The blood pressure of adults age 50 and older with general obesity in the community should be monitored regularly to identify patients with high blood pressure early.
